# A rare cause of high intestinal obstruction: Wilkie’s syndrome associated with a suprarenal abdominal aortic aneurysm

**DOI:** 10.1016/j.radcr.2025.04.084

**Published:** 2025-05-20

**Authors:** Chaimae Abourak, Siham Oukassem, Asmae Guennouni, Soukaina Bahha, Jamal El Fenni, En- Nouali Hassan

**Affiliations:** Department of Radiology, Hospital of Military Instruction Mohammed V (HMIMV), Mohamed V University, Rabat, Morocco

**Keywords:** Wilkie’s syndrome, Aneurysm, Abdominal aorta, High digestive obstruction

## Abstract

Wilkie’s syndrome, or superior mesenteric artery (SMA) syndrome, is a rare cause of duodenal obstruction due to extrinsic compression between the SMA and the abdominal aorta. We report the unusual case of a 45-year-old patient presenting with Wilkie’s syndrome associated with a suprarenal abdominal aortic aneurysm (AAA). The diagnosis was confirmed by an abdominal CT angiography, revealing a fusiform AAA of the suprarenal abdominal aorta, associated with a marked reduction in the aorto-mesenteric angle and compression of the third portion of the duodenum. A multidisciplinary approach was implemented, including initial conservative management with nasogastric decompression, followed by endovascular repair of the aneurysm. The postoperative course was favorable, with no complications. This case highlights a rare etiology and emphasizes the importance of precise diagnosis and a coordinated therapeutic approach, preventing severe complications.

## Introduction

Wilkie’s syndrome, also known as superior mesenteric artery (SMA) syndrome, is a rare cause of duodenal obstruction resulting from extrinsic compression between the SMA and the abdominal aorta [1,2]. Although various congenital and acquired etiologies have been documented, its presentation in association with an abdominal aortic aneurysm (AAA) is extremely rare. To our knowledge, no case has yet been reported in the literature where Wilkie’s syndrome occurs in the context of a suprarenal aneurysm without the aneurysm itself being the direct cause of duodenal compression.

In our case, the duodenal obstruction appears to result primarily from massive gastric distension, leading to an anterior extrinsic compression of the third portion of the duodenum. This situation was favored by a significant narrowing of the aorto-mesenteric angle, likely influenced by the presence of the suprarenal aneurysm.

This case highlights the diagnostic challenges associated with such atypical presentations and emphasizes the need for a multidisciplinary approach, including aneurysm repair and management of high intestinal obstruction. It also underscores the pivotal role of nasogastric decompression in breaking the obstructive feedback loop, which would not have been effective if the aneurysm had been the primary compressive agent.

## Case report

We report the case of a 45-year-old male patient with a medical history of chronic smoking, poorly controlled diabetes, and hypertension. For approximately one year, he had been experiencing chronic abdominal pain associated with loss of appetite. He presented to the emergency department due to worsening symptoms, including vomiting, cessation of stool and gas passage—clinical signs suggestive of a high intestinal obstruction.

On physical examination, the patient showed signs of dehydration and diffuse abdominal tenderness, without guarding or rigidity. Laboratory workup revealed significant hydroelectrolytic imbalances, notably hypokalemia and metabolic alkalosis, consistent with prolonged vomiting.

**Fig. 1:**
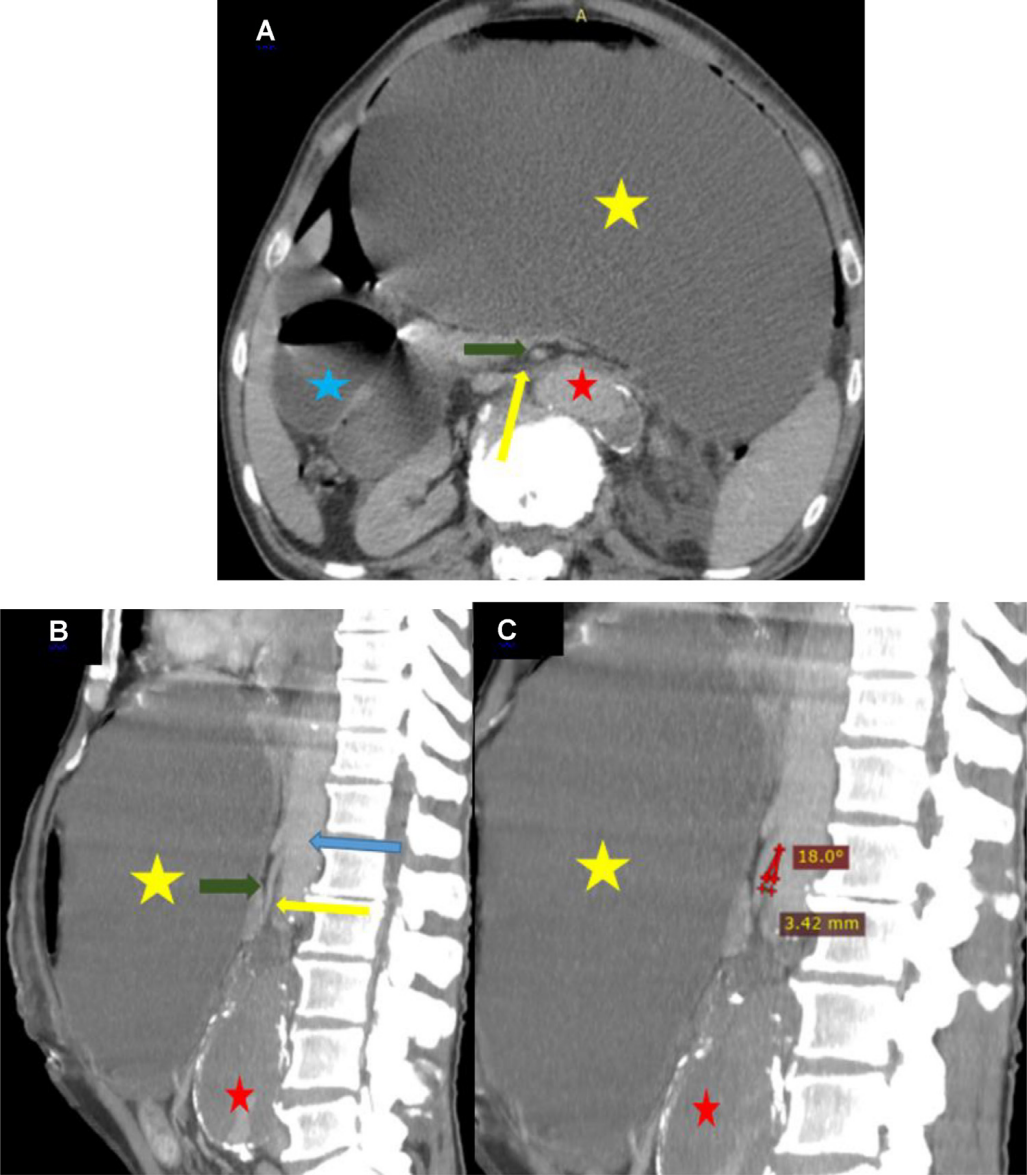
Abdominal CT Angiography: Axial view (a) and coronal reconstructions (b and c). The angle between the superior mesenteric artery (SMA) (green arrow) and the abdominal aorta (blue arrow) is markedly reduced to 18°, with a decreased aorto-mesenteric distance (3.42 mm). A fusiform suprarenal abdominal aortic aneurysm (red star) is present and may have contributed to the reduction of the aorto-mesenteric angle. This anatomical configuration leads to severe gastric and duodenal distension (yellow and blue stars), responsible for anterior extrinsic compression of the third portion of the duodenum (D3) (yellow arrow), which is trapped between the SMA and the aorta. The resulting duodenal obstruction is therefore secondary to distension-related mechanical compression rather than a direct mass effect from the aneurysm.

An urgent contrast-enhanced abdominal CT scan revealed a fusiform suprarenal abdominal aortic aneurysm (AAA) measuring 40×55 mm. A markedly reduced aortomesenteric angle (18°) was noted, along with a decreased distance (3.42 mm) between the posterior aspect of the superior mesenteric artery (SMA) and the anterior surface of the abdominal aorta. There was significant gastro-duodenal distension associated with air-fluid levels upstream of a narrowing at the third portion of the duodenum (D3), compressed between the SMA and the aorta (Fig. 1).

These findings were consistent with Wilkie’s syndrome (also known as superior mesenteric artery syndrome), where D3 is compressed due to a reduction in the aortomesenteric angle. In our case, the large suprarenal AAA appears to have indirectly altered the local anatomical relationships by narrowing the aortomesenteric angle, thereby promoting the development of the syndrome. The resulting gastric distension increased anterior pressure on D3, further aggravating the compression by mass effect and establishing a feedback loop of obstruction.

Initial management was conservative and included:

• Nasogastric decompression, which effectively interrupted the feedback loop by reducing intragastric pressure on the duodenum.
• Correction of hydroelectrolytic imbalances through intravenous rehydration with electrolyte supplementation.
• Nutritional support initiated via postpyloric feeding, using a high-calorie diet to bypass the obstruction and address the catabolic state.
• Postural maneuvers (e.g., knee-chest or left lateral decubitus positions) to relieve duodenal compression.
• Administration of prokinetic agents to stimulate gastrointestinal motility.

The patient showed rapid clinical improvement following gastric decompression, supporting the hypothesis of a functional and dynamic mechanism of duodenal compression, rather than a fixed mechanical obstruction caused by the aneurysm itself.

After clinical stabilization, the patient underwent endovascular aneurysm repair (EVAR), targeting the underlying anatomical factor. The procedure was uneventful, and the postoperative course was favorable, with no immediate or delayed complications. The patient was placed under regular follow-up.

## Discussion

Wilkie’s syndrome, also known as superior mesenteric artery (SMA) syndrome or aorto-mesenteric compression syndrome, is a rare condition caused by extrinsic compression of the third portion of the duodenum between the abdominal aorta and the SMA [[1], [2], [3], [4], [5]]. First described by Von Rokitansky in 1842, its clinical and radiological features were further detailed by Wilkie in 1927 in a series of 75 cases [1,2,4,5]. Fewer than 500 cases have been reported in the literature, with an estimated prevalence ranging from 0.013% to 0.3% [2,3,5].

This condition predominantly affects young females, with a mean age at diagnosis of approximately 23 years [2]. Therefore, the presentation in our patient, a 45-year-old male, represents an atypical clinical scenario.

The reduction in the aorto-mesenteric angle plays a central role in the pathophysiology of the syndrome. Under normal conditions, this angle ranges from 38° to 60°, and the aorto-mesenteric distance measures 10 to 28 mm. In Wilkie’s syndrome, the angle drops below 25°, and the distance may decrease to less than 8 mm [[1], [2], [3]], leading to extrinsic compression of the third part of the duodenum (D3) and subsequent obstructive symptoms.

Etiologies can be congenital—such as a short ligament of Treitz or a low origin of the SMA—or acquired, especially in the context of significant weight loss (e.g., anorexia nervosa, cancer cachexia, burns, or chronic illness), prolonged immobilization, or spinal surgery [[1], [2], [3]]. In approximately 40% of cases, no underlying cause is identified [1,4].

Our observation presents a previously unreported etiology: duodenal compression caused by a large suprarenal abdominal aortic aneurysm (AAA), which altered anatomical relationships and narrowed the aorto-mesenteric angle. To our knowledge, this pathophysiological mechanism has not been previously described.

Abdominal aortic aneurysms are defined as a permanent dilation exceeding 50% of the normal diameter, typically over 30 mm. They are more common in older men, smokers, and hypertensive individuals—features present in our patient [6]. The occurrence of Wilkie’s syndrome secondary to a suprarenal AAA represents a novel pathogenic mechanism, although duodenal compression due to other retroperitoneal masses—such as tumors, bulky lymphadenopathies, or chronic pancreatitis—has been reported, supporting the idea that any mass effect in this region may trigger a similar compressive syndrome [[7], [8], [9]].

Clinically, acute forms of Wilkie’s syndrome present with postprandial vomiting, high intestinal obstruction, and sometimes aspiration pneumonia [1,2,4]. Chronic forms are more insidious, characterized by epigastric pain, early satiety, weight loss, nausea, and gastroesophageal reflux [3,5]. In our patient, the sudden onset of acute obstruction on a background of chronic symptoms illustrates the progressive and dynamic nature of the obstruction.

The diagnosis is primarily radiological. A barium meal study may show proximal duodenal stasis. Contrast-enhanced abdominal CT is the modality of choice, providing precise assessment of anatomical relationships, including measurement of the aorto-mesenteric angle and distance, and identifying underlying etiologies [1,2,4]. In our case, CT angiography confirmed duodenal compression between the SMA and aorta, facilitated by a 55 mm fusiform aortic aneurysm, along with gastric dilatation and air-fluid levels.

Other mechanisms of duodenal obstruction described in similar settings include loss of mesenteric fat pad, upward traction on the duodenum, or congenital anomalies of intestinal rotation that narrow the aorto-mesenteric angle [1,3,8]. These can be exacerbated by associated conditions such as post-surgical spinal deformity or debilitating neurological syndromes [2].

The differential diagnosis includes other causes of extrinsic duodenal compression, such as retroperitoneal tumors, lymphadenopathy, chronic pancreatitis, pancreatic pseudocysts, post-surgical adhesions, or duodenal Crohn’s disease. However, the main differential remains idiopathic SMA syndrome, where no extrinsic mechanical cause is identified, despite similar imaging features [1,2,8,9].

Initial management is conservative, aiming to interrupt the obstructive cycle: nasogastric decompression, correction of fluid-electrolyte imbalances, post-pyloric enteral nutrition, positional therapy (left lateral or knee-chest), and prokinetic agents [[1], [2], [3]]. In our case, the rapid clinical improvement following decompression supported a dynamic rather than fixed mechanical process, reinforcing the diagnosis.

If conservative measures fail, surgical intervention is considered. Laparoscopic duodenojejunostomy remains the procedure of choice, with success rates exceeding 90% [1,2]. Other surgical options include SMA transposition or the Strong’s procedure.

In our patient, a multidisciplinary approach was adopted, including initial medical stabilization, followed by endovascular aneurysm repair (EVAR) of the abdominal aortic aneurysm. This targeted therapeutic strategy led to progressive resolution of the secondary Wilkie’s syndrome, validating the causal link between the aneurysm and duodenal obstruction.

## Conclusion

This case highlights a rare presentation of Wilkie's syndrome, where a suprarenal abdominal aortic aneurysm serves as the underlying cause of duodenal compression. This situation emphasizes the importance of a rigorous diagnostic approach, based on imaging, allowing differentiation between classic etiologies and unusual causes, such as AAA. It is crucial to promptly identify gastric distention as the true cause of the obstruction, which guides clinical management toward simple yet effective interventions, such as nasogastric decompression. The successful management, combining endovascular repair of the aneurysm and conservative treatment of the obstruction, underscores the importance of a multidisciplinary approach. This case enriches the literature by expanding the spectrum of causes of Wilkie's syndrome and could serve as a reference for future management of similar presentations.

## Ethics approval

Our institution does not require ethical approval for reporting individual cases or case series.

## Patient consent

Written informed consent was obtained from the patient(s) for their anonymized information to be published in this article.
